# Primary hepatic neuroendocrine tumor: A case report and literature review

**DOI:** 10.1097/MD.0000000000044492

**Published:** 2025-09-12

**Authors:** Chenglin Wang, Weiqiu Yu, Shasha Gao

**Affiliations:** a Department of Hepatobiliary and Pancreatic Medicine Center, WeiFang People’s Hospital, The First Affiliated Hospital of Shandong Second Medical University, Weifang, Shandong Province, China; b Department of Department of Ultrasound, Weifang Maternal and Child Health Hospital, Weifang, Shandong Province, China.

**Keywords:** hepatic primary, neuroendocrine tumor

## Abstract

**Rationale:**

Primary hepatic neuroendocrine tumors are extremely rare tumors originating from neuroendocrine cells. Due to the lack of neuroendocrine symptoms and specific imaging features, diagnosis is also challenging.

**Patient concerns:**

An elderly woman’s physical examination revealed a liver mass that had been present for 3 months. The patient presented with diffuse liver lesions deemed unresectable at the time of diagnosis. Interventional and chemotherapy treatment was performed after performing puncture pathology. However, the outcomes were unsatisfactory. This article reports a case of primary hepatic neuroendocrine tumor.

**Diagnoses:**

The pathological diagnosis was hepatic neuroendocrine tumor (G2 grade).

**Interventions:**

Implement CAPTEM combined with TACE treatment for patients.

**Outcomes:**

CAPTEM combined with TACE treatment was ineffective. The patient eventually lost access.

**Lessons:**

Primary hepatic neuroendocrine tumor surgical resection to obtain pathology is currently the best way for treatment and diagnosis. And call for the establishment of its own grading treatment guidelines, which are very important for evaluating the malignancy and prognosis of tumors.

## 1. Introduction

Revised version: Neuroendocrine tumors (NETs) commonly arise in the gastrointestinal and respiratory tracts; however, primary hepatic NETs are rare, representing approximately 0.3% of all NET cases.^[1]^ According to research statistics, as of 2020, only 150 cases of primary hepatic neuroendocrine tumors (PHNETs) have been reported in the literature.^[2]^ Moreover, due to its inconspicuous symptoms, nonspecific serology, and radiological manifestations that are very similar to other liver lesions, the diagnosis of PHNET is also relatively difficult.^[3]^ This report presents a case of primary hepatic neuroendocrine tumor and literature review, as follows.

## 2. Case data

**Figure 1.**
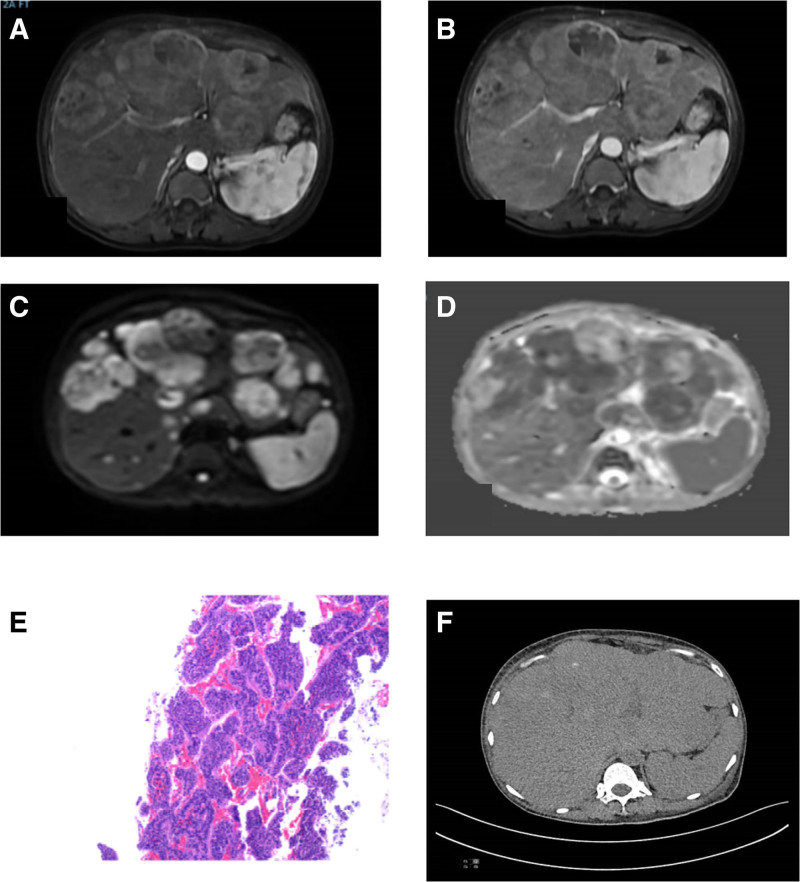
(A) Upper abdominal MRI plain scan + enhanced arterial phase. Multiple lesions of varying sizes, showing obvious uneven enhancement. (B) Upper abdominal MRI plain scan + enhanced venous phase. Decreased venous enhancement, with larger ones measuring approximately 10.3 × 7.7 cm in size. (C) Upper abdominal MRI plain scan + enhanced DWI. High signal intensity with significantly limited diffusion. (D) Upper abdominal MRI plain scan + enhanced ADC. Low signal. (E) Immunohistochemistry. HepPar-1(−), CD-10 (individual cells+), Glypican-3(−), CK19 (partially+), CK7(−), CK broad(+), CD56(+), Syn (+), CgA (slightly+), CD117(−), PAX-8(−), Ki-67 (index 20%), SSTR-2(3+). (F) Upper abdominal CT plain scan phase. Multiple lesions with slight high-density shadows within the lesions. ADC = apparent diffusion coefficient, CgA = chromogranin A, DWI = diffusion weighted imaging, MRI = magnetic resonance imaging.

The patient is a 69-year old female of Han ethnicity, who was admitted with the complaint of “liver mass found on physical examination for 3 months.” Previously healthy, denies any history of special or major illnesses or family history. No special treatment was administered before admission, and there were no obvious positive signs on physical examination upon admission. Laboratory test results were as follows: alanine aminotransferase 50 U/L, total bilirubin 31.7 µmol/L, direct bilirubin 15.8 µmol/L, tumor marker neurogenic specific enolase (NSE) 69.44 ng/mL. Whole abdominal MRI plain scan + enhancement (Fig. 1A–D): The liver volume increases, and diffuse nodules and masses of varying sizes are seen in the parenchyma. They show equal or slightly low signal on T1WI, slightly high signal on T2WI, high signal on DWI, and low signal on ADC. The arterial phase lesion shows obvious uneven enhancement on enhanced scan, and the venous and delayed phase enhancement decreases. The larger 1 is about 10.3 cm × 7.7 cm in size; Diagnosis: Multiple liver masses, considered malignant, hepatocellular carcinoma? Neuroendocrine carcinoma? The patient underwent gastroscopy and PET-CT, and no other obvious space occupying lesions were observed.

The patient had multiple intrahepatic masses and no opportunity for radical surgical resection. An ultrasound-guided liver mass biopsy was performed, and pathological examination showed (Fig. 1E): hepatic neuroendocrine tumor (G2 grade). Immunohistochemistry: HepPar-1(−), CD-10 (individual cells+), Glypican-3(−), CK19 (partial +), CK7(−), CK broad(+), CD56(+), Syn(+), CgA (slightly+), CD117(−), PAX-8(−), Ki-67 (index 20%), SSTR-2(3+). Administer CAPTEM regimen chemotherapy + TACE (4 mg of rituximab and 20 mg of pirarubicin), once every 3 weeks for local treatment. After more than 1 month of treatment, a follow-up abdominal CT scan showed no significant deposition of iodized oil in the liver tumor (Fig. 1F). The volume of the larger measurable tumor in the liver did not decrease significantly compared to before, and the NSE (80.91 ng/mL) increased compared to before, indicating poor treatment effect. The patient considered that this treatment plan was not suitable and went to a higher-level hospital for further treatment. Later, they contacted the patient again, but the patient refused to communicate about the progress of the condition and did not want to be disturbed, so they were lost to follow-up (clinical treatment timeline can be seen in Table 1).

**Table 1:** Clinical treatment timeline.

Point of time	Clinical events	Examination/treatment
Day 0	A liver lesion was found during the physical examination	Untreated, follow-up
Day 30	Be hospitalized	CT, MRI, Gastrointestinal endoscopy, tumor indicators
Day 34	Biopsy	Ultrasound guided liver mass biopsy and puncture
Day 36	Treatment	Intervention + chemotherapy
Day 69	Follow-up, disease progression	CT, tumor marker

CT = computed tomography, MRI = magnetic resonance imaging.

## 3. Discuss and conclusion

In previous studies, NETs mostly developed from enterochromaffin cells and were found in the gastrointestinal tract (55%), pancreas (2%), and bile duct (1%). However, they generally do not migrate to the liver.^[4]^ The origin of primary hepatic PHNETs remains unclear, with 3 main hypotheses proposed. First, PHNETs may arise from ectopic endocrine tissues within the liver, such as pancreatic or adrenal remnants. Second, they may derive from neuroendocrine differentiation of malignant hepatic stem cells. Third, and most widely accepted, is that PHNETs originate from neuroendocrine cells located in the epithelium of intrahepatic bile ducts.^[5]^ The 2025 edition of the Chinese Society of Clinical Oncology Guidelines for the Diagnosis and Treatment of Neuroendocrine Tumors (hereafter referred to as the “Chinese Neuroendocrine Tumor Guidelines”) states that approximately 10% of NETs are linked to genetic factors. PHNETs typically grow slowly and show no significant gender predilection. The average age at diagnosis ranges from 47 to 50 years. Lesions are usually solitary and mainly located in the right hepatic lobe. Due to the lack of distinctive imaging features, clinical manifestations, and serological markers, PHNETs are often misdiagnosed as other hepatic diseases.

PHNETs are classified as functional or nonfunctional based on their secretory activity.^[6]^ According to the Chinese Guidelines for Endocrine Tumors, most NETs are nonfunctional and may remain asymptomatic for years or even a lifetime. These tumors are often discovered incidentally during routine physical examinations or when patients seek medical care for symptoms such as abdominal distension and pain caused by the tumor’s mass effect. Functional NETs may present with clinical syndromes, including Cushing’s syndrome, carcinoid syndrome, or Zollinger-Ellison syndrome.^[7]^ The vast majority of PHNETs are nonfunctional. This may be due to defects that prevent activation of target organs, insufficient hormone secretion, or degradation of bioactive peptides and amines by liver enzymes before entering systemic circulation, thereby avoiding activation.^[8]^ The patient in this case was diagnosed with a nonfunctional PHNET. Imaging features of PHNET lesions are variable and often atypical. They may appear as nodular or cystic masses with well-defined margins or signs of peripheral invasion. Most lesions are hypervascular; about 26% show marked arterial phase enhancement followed by washout in the venous phase. Portal vein thrombosis is rare in these tumors. Because of these imaging characteristics, PHNETs are often misdiagnosed as hepatocellular carcinoma (HCC).^[9]^ Differentiating between these 2 conditions often requires comprehensive assessment, including medical history, laboratory tests, and imaging findings. For example, HCC is frequently associated with hepatitis B infection and cirrhosis. Serum alpha fetoprotein and protein induced by vitamin K absence or antagonist-II levels are typically elevated. Imaging of HCC often shows marked arterial phase enhancement with rapid washout in the venous and delayed phases, exhibiting a “fast in and fast out” pattern. Portal vein thrombosis is sometimes observed. Octreotide scanning is the preferred radiological examination for detecting NETs, given that the tumor expresses somatostatin receptor subtype 2, with a sensitivity of 75% to 95%. Serum tumor markers such as alpha fetoprotein and carcinoembryonic antigen have no practical diagnostic value for PHNETs. NSE is a commonly used serological marker for NETs. According to the 2023 European Guidelines for Neuroendocrine Tumors of the Digestive System, approximately 60% of patients have elevated chromogranin A and NSE in their blood. In this case, the patient’s NSE level was elevated. Pathology plays an important role in the diagnosis of PHNET. Currently, the histopathology of PHNET can refer to other NETs in the “Chinese Gastrointestinal Pancreatic Neuroendocrine Tumor Expert Consensus (2022 Edition)”; chromogranin A, Syn, and CD56 are currently recognized as specific immunohistochemical markers for diagnosing NET, and this case is consistent; The diagnostic grading of NETs has clear reference standards, which will not be elaborated here. Therefore, tumor markers, imaging, and pathology cannot distinguish whether it is primary or secondary, CT, MRI, and PET. After careful examination such as gastroscopy and colonoscopy, rule out. The diagnosis of PHNETs primarily depends on definitive pathological findings, while preoperative examinations mainly aim to exclude extrahepatic primary sources. Therefore, the diagnosis of PHNET is often considered one of exclusion. The Chinese Guidelines for Endocrine Tumors emphasize that a notable characteristic of NETs is their potential for recurrence and metastasis. Lesions at different anatomical sites and time points may display variations in pathological differentiation, grading, or molecular alterations. The most common scenario involves the progression of low-grade tumors to high-grade tumors. Therefore, for metastatic endocrine tumors, multisite and longitudinal sampling is recommended to guide optimal treatment strategies.

The treatment strategy for PHNETs parallels that of HCC, with surgical resection being the preferred option. The choice of surgical technique depends on the tumor’s size and location, and achieving an R0 resection with negative margins, along with appropriate lymphadenectomy, is considered optimal. The reported 5-year survival and recurrence rates following surgery are approximately 74% and 18%, respectively. For patients who are not candidates for surgery, systemic therapies are largely extrapolated from treatments for other NETs. Despite limited evidence, transcatheter arterial chemoembolization, systemic chemotherapy, and somatostatin analogs have not demonstrated clear long-term survival benefits. Given that PHNETs are highly vascular tumors sensitive to ischemia, TACE may be recommended for unresectable or recurrent cases. However, current literature indicates that TACE primarily provides favorable short-term therapeutic effects.^[10]^ The patient exhibited minimal deposition of iodized oil following transcatheter arterial chemoembolization, resulting in a poor therapeutic response. Regarding immunotherapy, the 2023 European Guidelines for Digestive System Neuroendocrine Carcinomas recommend that MSI/dMMR testing should be performed for digestive system NEC, and drugs should be selected based on the test results. However, there is no first-line recommendation for PHNET. For unresectable PHNETs with unfavorable treatment outcomes and absence of extrahepatic metastases, liver transplantation may be considered as a treatment option.^[11]^ However, the widespread application of liver transplantation is limited by high costs and scarce donor organs. The prognosis is determined by the functional status of the tumor, pathological grading and differentiation, staging, and treatment method.

The prognosis of PHNETs is influenced by multiple factors, and the overall survival rate remains poor. Currently, effective prognostic markers are lacking. However, recent studies have offered promising research directions. Some evidence suggests that tumor-associated inflammation significantly impacts DNA damage, gene mutation, angiogenesis, tumor proliferation, invasion, and metastasis. Cancer-related inflammation plays a critical role in both prognosis and postoperative recovery. Systemic inflammation affects the tumor microenvironment and may regulate immune surveillance and therapeutic response. Therefore, inflammation-based biomarkers are expected to become important predictors of surgical outcomes and long-term prognosis.^[12]^ The modified Glasgow Prognostic Score, a scoring system based on systemic inflammation, has emerged as a valuable prognostic tool across various cancers. This simple, noninvasive system has been validated in prostate, gynecological, lung, colorectal, and breast cancers, showing consistent associations with poor survival.^[13]^ In addition, dysregulation of Ca^2+^ signaling has been implicated in the progression of diverse cancer pathways. This dysregulation may be tissue-specific, context-dependent, or common across cancer types. Targeting Ca^2+^ regulation thus represents a promising strategy for developing future context-specific cancer therapies. These findings provide a useful framework for further evaluating the prognosis of PHNET.^[14]^

PHNETs lack distinctive oncological features, complicating preoperative diagnosis. Thus, comprehensive preoperative examination and evaluation are necessary. Surgical resection remains the optimal approach for both diagnosis and treatment by allowing pathological confirmation. Although the World Health Organization has established clear grading guidelines for gastrointestinal and pancreatic NETs, no unified grading system or standardized treatment protocol exists specifically for PHNETs. Consequently, most clinicians apply the classification criteria for gastrointestinal NETs to PHNETs. In this case, the patient was managed according to the grading and treatment guidelines for gastrointestinal and pancreatic NETs; however, the therapeutic response was suboptimal. This outcome may stem from differences in cellular origin and physiology between PHNETs and other NETs. Therefore, it is imperative to develop specific grading and treatment guidelines for PHNETs. Such guidelines are critical for accurately assessing tumor malignancy and prognosis.

## Author contributions

**Data curation:** Weiqiu Yu.

**Funding acquisition:** Weiqiu Yu, Shasha Gao.

**Resources:** Shasha Gao.

**Writing – original draft:** Chenglin Wang.

**Writing** – **review & editing:** Chenglin Wang.
